# Clonal Haematopoiesis in Type 2 Diabetes: A Review of Mechanistic Links With Inflammation and Cardiovascular Disease

**DOI:** 10.1002/dmrr.70137

**Published:** 2026-02-10

**Authors:** Ludovica Migliozzi, Marella Marassi, Mattia Albiero, Gian Paolo Fadini

**Affiliations:** ^1^ Department of Medicine University of Padova Padova Italy; ^2^ Veneto Institute of Molecular Medicine Padova Italy

**Keywords:** cardiovascular disease, clonal haematopoiesis, diabetes, inflammation, metabolism

## Abstract

Clonal haematopoiesis (CH) has been recognized as an important interface between chronic inflammation, metabolic dysfunction, and the heightened cardiovascular risk observed in type 2 diabetes (T2D). CH arises from somatic mutations that give haematopoietic stem cells a competitive advantage and drive the expansion of pro‐inflammatory myeloid lineages. These mutant cells exhibit amplified IL‐1β production, enhanced NLRP3‐inflammasome activity, and increased chemokine signalling, thereby accelerating atherosclerosis, insulin resistance, and vascular inflammation. At the same time, metabolic disturbances characteristic of T2D promote clonal expansion by weakening normal stem cell fitness and modifying epigenetic regulation. This creates a self‐reinforcing loop in which inflammation and metabolic stress sustain CH growth, while CH‐derived myeloid cells worsen systemic and tissue‐level inflammation. Such interactions may contribute not only to excess coronary disease and heart failure but also to microvascular complications through heightened myelopoiesis, neutrophil activation, and dysfunctional stem‐cell mobilisation. This review integrates epidemiological, mechanistic, and experimental findings to present a unified model in which CH and diabetic metabolic stress act synergistically. We explore the shared pathways (niche remodelling, inflammatory memory, and metabolic rewiring) that underpin this bidirectional relationship and discusses how CH may differentially shape cardiometabolic outcomes. Future perspectives in this field are also discussed, including refining CH detection, integrating clone characteristics into risk stratification, and developing targeted therapies that disrupt the metabolic and inflammatory circuits supporting clonal expansion. Anti‐inflammatory strategies and metabolic modulators may ultimately provide personalised approaches to reduce CH‐associated cardiovascular risk in T2D.

## 1 Introduction

Despite advancing knowledge in the mechanisms linking type 2 diabetes (T2D) to excess cardiovascular disease (CVD), people with diabetes are still exposed to an increased cardiovascular mortality. Chronic low‐grade sterile inflammation is an established proposed mechanism of vascular complications in T2D; however, the precise cellular and molecular triggers of this inflammatory burden are not fully defined. Traditional risk factors such as hyperglycemia, dyslipidemia, and obesity explain only in part the increased CVD incidence, leaving a knowledge gap that suggests additional, unrecognised contributors. In this context, clonal haematopoiesis (CH) of indeterminate potential has recently emerged as a possible unifying factor for the interplay among inflammation, diabetes, and CVD.

## 2 Definition of Clonal Haematopoiesis

The term CH ‘of indeterminate potential’ was first introduced by Steensma et al. in 2015 [1] to describe the presence of somatic mutations in haematopoietic stem cells, canonically associated with haematologic malignancy, that drive the expansion of clones although in the absence of haematological neoplasms or cytopenias [2]. The World Health Organization (WHO) and the International Consensus Classification (ICC) define CH as the presence of one or more acquired somatic mutations in genes associated with myeloid malignancies, detected at a variant allele frequency (VAF) of ≥ 2% [3]. Another feature of CH is the low rate of progression to overt neoplasia (0.5%–1% per year) [1].

The estimated prevalence of CH varies depending on the gene sequencing approach, although with the advent of more sensitive technologies, CH is detected in increasingly larger proportions of the general population. The main sequencing approaches include whole‐genome sequencing (WGS), whole‐exome sequencing (WES), targeted sequencing, and error‐corrected sequencing [4]. CH is estimated to be present in approximately 5% of adults aged 40–70 years, with prevalence rising to 10%–20% in individuals over 70 years of age [5]. CH–related mutations have been identified in 72 genes, with the most frequently affected being *DNMT3A*, *TET2*, *ASXL1*, and *JAK2*, which are typically implicated in myeloid malignancies [6].

The specifier ‘of indeterminate [oncogenic] potential’ has traditionally referred to the slow and uncertain neoplastic evolution of clonal haematopoiesis (CH), pending acquisition of additional oncogenic mutations. However, such *indeterminate potentiality* has been increasingly questioned, as growing evidence shows that CH confers substantial risks for morbidity and mortality, even in the absence of overt haematologic diseases.

### 2.1 Impact of CH on Haematological and Non‐Haematological Diseases

Although candidate driver mutations associated with CH and haematological malignancies often overlap [7], CH is mostly asymptomatic without alterations in the blood cell counts and rarely progressive. The absolute risk of developing haematological malignancies among individuals with CH mutations is very low, approximatively 0.5% per year [7]. However, recent studies have reported that specific CH features increase the risk of malignant transformation [8], such as the presence of TP53 mutations, VAF > 10%, specific variants, and cytopenias. Moreover, haematopoietic stressors, such as inflammation, chemotherapy and ribosomal stress, can increase the risk of myeloid malignancies.

Nevertheless, an approximately 40% increase in mortality has been reported in individuals with CH [9], and this excess risk primarily arises from its association with age‐related disorders [10]. Among these, cardiovascular disease (CVD), including coronary artery disease (CAD), atherosclerotic cardiovascular disease (ASCVD), and myocardial infarction, represent the leading causes of death in individuals harbouring CH mutations. Despite acknowledging inter‐study heterogeneity in clone size, study populations, and sequencing methods, a recent meta‐analysis confirmed that CH increases the risk of both all‐cause mortality (HR 1.34; 95% CI, 1.19–1.50) and cardiovascular events (HR 1.23; 95% CI, 1.07–1.41) [4]. Consistently, individuals with established ASCVD carrying any CH mutation had a 23% increased risk of mortality, whereas those with larger clones (VAF > 10%) faced a 34% higher risk [11]. Notably, in another study, patients with CAD carrying CH mutations exhibited a 70% higher 3‐year mortality risk than matched CAD controls [12]. These adverse clinical outcomes are thought to be mediated by an enhanced inflammatory state [13]. This concept is supported by an *Ldlr* ^−/−^ murine model of atherosclerosis, in which haematopoietic *Tet2* loss was sufficient to aggravate atherogenesis via augmented cytokine production [14]. Furthermore, recent findings indicate that CH mutations contribute to other organ dysfunctions. WGS and WES studies have shown that individuals with severe chronic obstructive pulmonary disease (COPD) are more likely to harbour CH. Consistently, *Tet2* loss in haematopoietic murine cells exacerbates emphysema and promotes pulmonary inflammation [15]. Additionally, a meta‐analysis of data from three population‐based cohorts reported an overall 26% higher risk of acute kidney injury (AKI) associated with CH, with individuals harbouring large CH having a 2.4‐fold higher risk of impaired renal recovery [16]; in parallel, epidemiological and experimental studies also indicate CH as a predictor of renal function decline and adverse outcomes in chronic kidney disease [17, 18].

Individuals with CH also display an increased risk of developing solid tumours, including lung, colorectal, prostate, and breast cancer [19, 20, 21]. The proposed mechanisms include impaired immune surveillance and alterations of the tumour microenvironment [22]. For example, a recent study reported that in individuals with CH and lung cancer, TET2 mutations promoted CH clone‐derived monocyte migration into lung tumour tissue, promoting a remodelling of the tumour immune microenvironment [23]. Furthermore, chemotherapy or radiation therapy for solid tumours can promote the emergence of CH, which in turn may further increase the risk of haematologic malignancies [24].

## 3 Evidence Supporting an Association Between CH and Diabetes

Type 2 diabetes (T2D) is a multifactorial disease and a growing concern for healthcare systems worldwide. Individuals with T2D have a significantly higher risk of developing CVD and atherosclerosis, which are major contributors to excess morbidity and mortality [25]. T2D is also considered a pro‐ageing condition, as it accelerates ageing‐associated detrimental processes, ultimately leading to multi‐organ dysfunction that shortens life expectancy [26]. Given that CH is associated with CVD and biological ageing, several attempts have attempted to investigate the bidirectional link between CH and T2D often with inconclusive and conflicting results [7, 27]. However, the interpretation of cross‐sectional study warrants caution as their design preclude inference of causality. Indeed, it would be important to address whether CH is associated with the development of T2D or whether T2D favours the onset of CH. Initial evidence from DNA arrays reported the presence of clonal mosaicism in the peripheral blood of 7659 individuals with T2D, and carriers showed a 71.4% prevalence of vascular complications compared to 37.1% among non‐carriers [28] and *TET2 loss of* function has been observed in diabetic patients [29].

More informative evidence on CH as a potential risk factor for T2D comes from a longitudinal study of 17,637 participants, which identified CH carriers harbouring a 23% increased risk of developing T2D compared with non‐carriers over a median follow‐up of 9.8 years, particularly when mutations occurred in DNMT3A, TET2, ASXL1, and JAK2 [30]. Similarly, a Korean study reported that CH synergised with elevated LDL cholesterol to increase the risk of new‐onset T2D, which was approximately twofold higher in individuals with both CH and hypercholesterolaemia compared with those lacking either condition [31].

Such clinical findings have been recapitulated in murine models, where *Tet2*–driven CH aggravated insulin resistance and increased fasting blood glucose levels, likely via a heightened IL‐1β production in adipose tissue [32]. Similarly, obese mice with CH mutations displayed an expansion of mutant cells and obese mice transplanted with Tet2‐KO bone marrow had enhanced production of pro‐inflammatory myeloid cells [33].

On the other hand, metabolic dysfunctions may in turn favour CH development and expansion. A longitudinal study of CH trajectories over 20 years in individuals with obesity revealed a correlation between CH‐clone growth rate and markers of metabolic abnormality, such as low HDL and HOMA‐IR as a surrogate for insulin resistance [34]. Consistent with this, a Mendelian randomisation analysis using data from the UK Biobank and the FinnGen consortium supported a causal role of T2D in TET2‐CH development [35]. Mechanistic insights are provided by a murine model of genetic obesity, where an accelerated expansion of CH‐mutant clones was driven by obesity‐related remodelling of bone marrow (BM) microenvironment [33]. Furthermore, hyperglycemia in obese mice has been shown to induce aberrant calcium signalling and accumulation of the metabolite itaconate, which fosters clonal expansion via the inhibition of Tet‐mediated DNA methylation [33]. Similarly, increased glucose levels impair AMPK‐dependent phosphorylation of Tet2, leading to epigenetic dysregulation [29].

**FIGURE 1:**
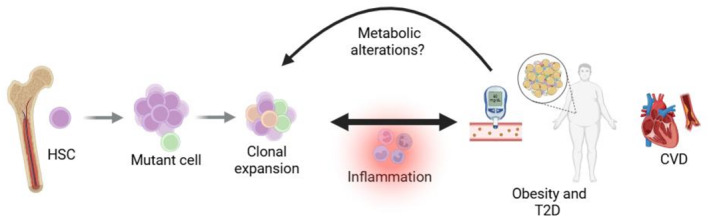
Bidirectional relationship between CH and T2D. CH increases the risk of developing T2D, obesity and its associated CVD possibly through inflammation. In parallel, metabolic alterations and inflammation may further exacerbate the expansion of mutant clones. The proposed mechanism is based on studies in both mice and humans.

Altogether, this evidence points to a bidirectional relationship between CH and T2D. On the one hand, CH may promote the onset and progression of diabetes, while on the other hand, metabolic disturbances associated with diabetes appear to accelerate the expansion of mutant clones and amplify the pathogenic effects of CH [34] (Figure 1).

Although the precise mechanisms underlying this reciprocal interaction remain unclear, inflammation is emerging as the likely converging pathway through which diabetes and CH reciprocally potentiate each other's detrimental effects [32].

Furthermore, metabolism may also play a direct role in regulating HSC fate. For instance, *TET2* enzymatic activity depends on α‐ketoglutarate, a key intermediate of the tricarboxylic acid (TCA) cycle, highlighting the close links between CH mutations and cellular metabolism [36]. This connection suggests that metabolic rewiring in HSCs may modify CH progression and its impact on CVD and T2D. Conversely, CH mutations fuel metabolic stress by enhancing glycolysis and ROS production [37].

Finally, alterations in gut homoeostasis represent another potential player involved in CH onset and regulation. Age‐related intestinal changes promote the dissemination of bacterial products, such as ADP‐heptose produced by Gram‐negative bacteria, which enhance HSC proliferation [38], while microbiota‐derived butyrate, by modulating the BM niche, regulates HSC fate decisions to accommodate haematopoietic stress [39]. Increased intestinal permeability is a prototypical feature of metabolic inflammation [40]. Neutrophils respond to microbiota alterations by worsening intestinal permeability, which links meta‐inflammation to the development of dysmetabolism [41]. Therefore, alterations in the gut microenvironment may trigger haematopoietic stress leading to CH and, at the same time, promoting T2D. On the other hand, CH‐driven somatic mutations empower a pro‐inflammatory myeloid skewing, which aggravates intestinal dysfunction, potentially fostering a vicious cycle [42].

### 3.1 CH and the Development of Chronic Diabetic Complications

The burden of diabetes is not only explained by its high prevalence, but also by the severity of its multiple chronic complications [43].

CH mutations are strongly associated with CVD, including atherosclerosis and heart failure, providing a further mechanistic link between T2D and the development of macrovascular complications [28], through multiple mechanisms that are still only partially understood. Supporting this, in a prospective UK Biobank analysis among over 20,000 T2D individuals, CH was associated with a 21% increased risk of CVD, a 18% increased risk of coronary heart disease, and a 73% increased risk of heart failure. Notably, CH‐associated CVD risk was independent of conventional health indicators such as BMI, blood glucose, blood pressure, and lipids [44]. Similarly, Zeng et al. found that CH mutations synergistically interacted with accelerated biological ageing substantially increasing CVD risk in T2D individuals [45]. Multiple mechanisms have been reported to account for these findings. In the aforementioned study by Zeng, the observed detrimental effects appear to be mediated, at least in part, by inflammatory pathways, including neutrophil degranulation and extracellular matrix production, which amplify inflammation and tissue damage.

Hyperglycemia promotes the activation of neutrophils through the alarmin S100A8/A9, which in turn imposes replicative pressure on HSPCs and increases the production of monocytes and neutrophils. This heightened inflammatory state ultimately promotes atherosclerosis [46]. Similarly, in obesity, S100A8/A9 induces the expression of IL‐1β in adipose tissue macrophages, which boost myelopoiesis by stimulating haematopoietic progenitors in the bone marrow, generating a pro‐inflammatory milieu which can sustain cardiovascular risk [47].

Recent evidence has associated CH with an increased incidence of microvascular complications. WES analyses of 21,784 participants with T2D and CH from the UK Biobank, followed over a median of 13.0 years, revealed a 23% higher risk of diabetic retinopathy and a 26% higher risk of kidney disease, but no increased risk of neuropathy, compared with those without CH [48].

Overall, multiple studies suggest a role for inflammation as a spillover of CH, which may contribute to the exacerbation of both macrovascular and microvascular complications in individuals with T2D.

## 4 Pathophysiological Mechanisms of the Association Between CH and Diabetes

### 4.1 Pro‐Inflammatory Drift of Immune Cells

Mounting evidence supports that CHIP exacerbates inflammatory response, which in turn constitutes a plausible key mediator of CH‐associated adverse effects (Table 1).

**TABLE 1:** CH‐related genes and related inflammatory mechanisms.

Affected gene	Inflammatory phenotype	T2D features/complications	References
TET2	NLRP3‐inflammasome	Adipose tissue dysfunction, insulin resistance, liver glucose production, atherosclerosis	[13, 14, 49]
	IL‐1β driven macrophage activation		
JAK2	Neutrophil activation, NETosis	Thrombosis, atherosclerosis	[49, 50]
	IL‐1β driven inflammasome activation		
DNMT3A	IL‐6, TNF‐α, IL‐1 macrophage activation	Metabolic dysfunctions, weight gain, fibrosis	[51, 52]
ASXL1	C‐reactive protein	CVD dysfunctions	[53]

For example, the mechanisms potentially underlying the higher CVD risk—up to twofold for CAD – reported among individuals with TET2‐dependent CH [13] have been unravelled in murine models of atherosclerosis. In these models, haematopoietic *Tet2* loss accelerated atherogenesis via increased IL‐1β secretion through the NLRP3‐inflammasome pathway [14] and augmented CXC chemokine expression in macrophages, promoting their recruitment to lesion sites [13]. Similarly, in mice, Jak2 mutations enhanced IL‐1β production through inflammasome activation, leading to an increased burden of inflammatory macrophages within atherosclerotic lesions [49]. Offering a mechanistic explanation for the contribution of CH to such organ dysfunctions, macrophages from Tet2 and Jak2 mutant mice have been shown to localise in the kidney in response to insults, where they mediate an aberrant inflammatory response resulting in exaggerated fibrosis and impaired recovery [16]. *Tet2* KO haematopoietic stem and progenitor cells (HSPCs) exhibited resistance to apoptosis and enhanced production of IL‐6 [54] and when exposed to IL‐1β empower a myeloid skewing [42], while *Tet2* KO mice treated with IL‐1β displayed increased pro‐inflammatory macrophages [55]. Likewise, loss of *Dnmt3a* in myeloid cells promoted atherosclerosis in mice and increased inflammation in macrophages in vitro [56]. Single cell RNA sequencing of circulating cells from people with heart failure carrying *Dmt3a* mutations revealed altered gene signatures in monocytes, macrophages, NK cells, and CD4^+^ T, which amplifies inflammation contributing to cardiac dysfunction and fibrosis [57] by paracrine activation of cardiac fibroblasts [52] Mutations in ASXL1 also contribute to adverse clinical outcomes by dysregulating innate immunity although the inflammatory profile differs from that driven by *Tet2* and *Dnmt3a* mutations. For example, people with atherosclerosis and ASXL1‐CH exhibit elevated levels of C‐reactive protein but not of IL‐1β [53].

Human studies have confirmed increased pro‐inflammatory cytokines in individuals with CH. Individuals with TET2‐CH exhibited significantly higher levels of interleukin‐8, the prototypical CXC chemokine in humans [13]. Interestingly, WES data from the UK Biobank showed that among 417,570 participants, 10.6% experienced CVD events, and this was associated with variants in inflammasome‐related genes [58]. A recent meta‐analysis of the UK Biobank, the Atherosclerosis Risk in Communities Study, and the Cardiovascular Health Study, identified an overall 26% increased risk of acute kidney injury in individuals with CH, which was triggered by renal macrophage inflammation [16].

The role of neutrophils in CH‐driven inflammation has gained increasing attention. One mechanism by which neutrophils clear pathogens is the release of neutrophil extracellular traps (NETs), eventually followed by cell death through NETosis. NETs are web‐like structures formed by DNA, histones and proteins that physically entrap and degrade pathogens. In addition to microbial stimuli, NET formation can be triggered by endogenous factors, such as in hyperglycemia or stress conditions [59]. However, uncontrolled NET release has detrimental consequences and contributes to organ dysfunction by amplifying the inflammatory response, accumulating within atherosclerotic plaques, and promoting fibrin deposition and platelet aggregation in thrombi [60]. Moreover, NETs worsen tumour outcomes by promoting cancer cell proliferation or by accumulating at metastatic sites; cancer cells also elicit signals that influence neutrophil production and function [61].

In the context of CH, mouse harbouring *JAK2* mutation showed increased formation of NETs, which aggravated thrombosis. Similarly, neutrophils from patients with myeloid disorders carrying *JAK2* mutations displayed enhanced NET formation [50]. Interestingly, human neutrophils carrying *Tet2* mutations had a preferential differentiation towards granulocytic bias. In particular, mutated neutrophils produce more stable NETs that trigger vascular occlusion and platelet aggregation, contributing to cardiovascular pathologies [62]. As previously mentioned, inflammatory activation linked to neutrophil degranulation has been identified as a potential link between biological ageing and CVD in individuals with diabetes [45] and could represent a target for cardiometabolic diseases [63]. Excessive or dysregulated NETs and NETosis have been extensively reported to play a role in the development of diabetes and its complications [41, 60]. Altogether, the evidence that CH mutations activate neutrophils, particularly by promoting NET formation, suggests an additional mechanism by which CH‐driven inflammation may contribute to diabetes and its complications.

#### 4.1.1 Alterations of the BM Niche Homoeostasis in the Contest of CH

Haematopoietic stem progenitor cells (HSPCs) are nested in the bone marrow for maintaining haematopoiesis, but they also migrate into the circulation through a process known as mobilisation, which is critical for tissue homoeostasis, immune surveillance and stress‐elicited for extramedullary haematopoiesis. In individuals with diabetes, circulating CD34^+^ HSPCs are reduced by approximately 30%–40% compared with healthy controls [64] and this reduction is associated with adverse cardiovascular outcomes [65] and with micro‐ and macrovascular complications [66]. Reduced levels of circulating progenitor cells in individuals with T2D were associated with cumulative progression of microangiopathy of 9.5%, and predicted microvascular outcomes [67]. A recent study with a median follow up of 6.7 years, reported that individuals with T2D and low HSPC levels had a higher incidence of nephropathy onset or progression [68]. Reduced HSPC counts have been attributed, in part, to impaired release into the peripheral blood caused by defective BM mobilisation, termed ‘diabetic stem cell mobilopathy’ [69], which arises from structural and functional alterations in the BM. Diabetes is associated with BM microangiopathy, autonomic neuropathy, inflammation, and stromal changes, including reduced osteoclast abundance and increased BM fat [70]. Impaired mobilisation has detrimental clinical consequences, such as poorer outcomes after stem cell transplantation, and is linked to higher risks of cardiovascular disease and death [70]. Recent evidence suggests that, in diabetes, myelopoiesis and mobilopathy are intrinsically linked and jointly contribute to adverse long‐term cardiovascular outcomes [71, 72]. Hyperglycemia and adipose tissue–derived signals independently promote a myeloid‐biased differentiation of HSPCs in the diabetic BM [47]. As a result, pro‐inflammatory neutrophils and monocytes spread in the circulation, driving diabetic vascular complications [46]. Within the BM microenvironment, macrophages of the haematopoietic niche release IL‐6 family cytokine Oncostatin M (OSM), which induces CXCL12 expression in stromal cells, enhancing HSPC retention at the expense of mobilisation [73]. Consistently, individuals with CAD and low HSPC levels exhibit elevated levels of IL‐6, which correlates with an increased incidence of cardiovascular events [74].

Most common CH‐associated genes, such as Dnmt3a, Aslx1 and Tet2, regulate DNA methylation and chromatin accessibility and drive a skewed differentiation of HSPCs towards a myeloid bias at the expense of lymphoid lineages. For instance, loss of *Tet2* function opens chromatin at PU.1‐driven enhancers, favouring monocyte/macrophage lineage differentiation [55], accelerates atherogenesis in mice [13] and promotes HSPC self‐renewal reinforcing the myeloid bias in a sort of vicious cycle [75]. Similarly, loss of *Dnmt3a* results in abnormal myeloid cell growth [76]. These findings may explain why CH mutations affect the activity of myeloid cells, in particular neutrophils and macrophages, which are central mediators of cardio‐metabolic diseases [77]. HSPCs purified from elderly people display upregulation of gene programs associated with myeloid differentiation, a finding confirmed by clonal composition analyses of HSPC pools in ageing mice [78]. Moreover, higher circulating levels of S100A8 and other inflammatory mediators in frail older people are accompanied by HSPC deficits, both contributing to CVD events [79].

Whether CH is a cause or amplifier of mobilopathy is still unknown. Since CH drives a myeloid‐biased differentiation of HSPCs and myelopoiesis is known to induce mobilopathy, it is reasonable to hypothesise that CH could contribute to mobilopathy, which in turn leads to impaired vascular repair and defective immune surveillance. Consequently, the combination of enhanced myelopoiesis and mobilopathy compromises these functions. However, mobilopathy may also arise from other factors, such as gluco‐ and lypo‐toxicity that disrupt BM structure and function [71]. Thus, individuals with mobilopathy may not necessarily harbour CH‐associated mutations. Inflammation appears to be the common underlying mechanism linking these two conditions. Nevertheless, additional CH‐associated mutations may affect stem cell self‐renewal, migration, differentiation, and BM localisation, further influencing haematopoietic and immune homoeostasis.

**FIGURE 2:**
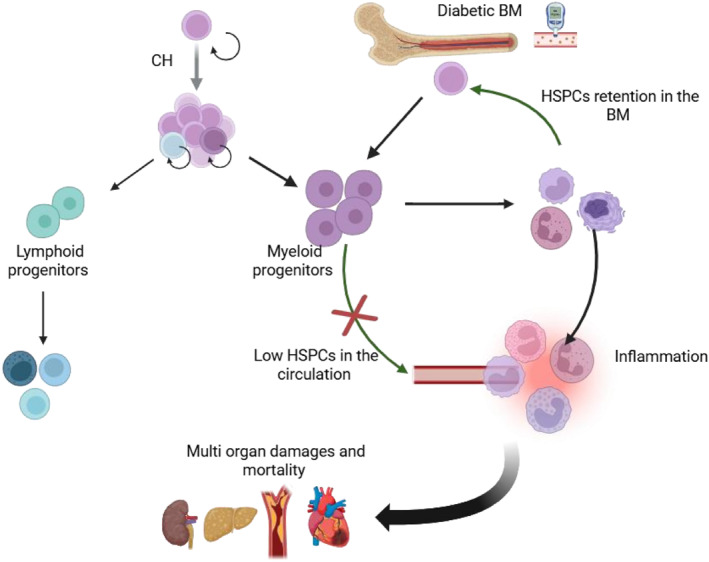
Mechanistic link between CH, myeloid bias, and mobilopathy in diabetes. Both CH and diabetes promote a myeloid‐biased differentiation of HSPCs, resulting in the production of pro‐inflammatory cells that circulate systemically and contribute to multi‐organ damage and increased mortality. In addition, pro‐inflammatory neutrophils and macrophages signal within the bone marrow to retain HSPCs, thereby impairing their mobilisation into the circulation. The green arrow indicates that the causal link has been obtained only from murine evidence, while the black represents both human and murine data.

On this basis, reduced circulating HSPC levels, together with enhanced myelopoiesis, may represent an unappreciated mechanistic link between CH and diabetic BM pathology (Figure 2). HSPC defects and CH are both tightly connected with cardiovascular outcomes in diabetes [71]. Moreover, the association of CH with cardiovascular disease and ageing supports these shared pathophysiological mechanisms bridging diabetes with age‐related diseases.

## 5 Future Perspectives and Diagnostic‐Therapeutic Implications

CH is an increasingly recognised contributor to age‐related diseases, particularly CVD although the precise mechanisms by which CH mutations increase cardiovascular risk remain incompletely understood. While CH of accumulate progressively with age, most individuals die from cardiovascular events before developing haematologic malignancies.

More recently, CH has also been identified as a risk factor for T2D and its associated cardiovascular complications. Indeed, carriers of CH mutations, particularly in DNMT3A, TET2, ASXL1, and JAK2, display a higher risk of developing both T2D [30], and CVD [44]. In turn, metabolic alterations and associated chronic inflammation appear to exacerbate the effects of CH. In this manuscript, we have comprehensively reviewed available evidence on a mutual interplay between T2D, CH and CVD in clinical research, discussed how these observations have been recapitulated in pre‐models, and recognised inflammation as a potential mechanistic bridge between these conditions.

Important gaps in knowledge remain. For example, it is still unclear whether the degree of hyperglycemia aggravates CH‐related outcomes, or whether the presence of diabetic complications further increases the risk of CH‐associated mortality. These questions warrant future investigation.

In this framework, the concept of inflammatory memory provides another layer of explanation. Upon exposure to inflammatory stressors, HSPCs retain a sort of memory which features persistent epigenetic remodelling leading to functional defects. These modifications increased myeloid differentiation, and sustained inflammation that may foster CH development [80].

Thus, the concomitant presence of CH mutations and inflammatory and metabolic cues may synergistically exacerbate the dysfunctions observed in T2D. Supporting this, CH not only increases mortality in individuals with T2D but also enhances susceptibility to Tet2 clonal expansion [35].

Although key mechanistic gaps remain, multiple studies have demonstrated that CH mutations increase the risk of vascular events through heightened inflammation, remarking once more the central role of both local and systemic immune activation in driving atherosclerosis and T2D.

Therefore, therapeutic strategies can be designed to tackle the inflammatory mediators or target the mutant clones. First, the finding that clone size increased with age in individuals with obesity, but not in those who underwent bariatric surgery [34], may suggest that achieving a profound metabolic reprogramming—as occurs following surgery—may constitute a potential strategy to attenuate the age‐related clonal expansion.

Moreover, anti‐inflammatory therapies have already shown promising results in CVD, as demonstrated in the CANTOS trial, where IL‐1β inhibition with canakinumab reduced cardiovascular events by 62% in people with Tet2 mutations [81]. Moreover, colchicine attenuated atherosclerosis in Tet2 KO mice and reduced the risk of myocardial infarction in human Tet2 carriers [82]. In parallel, systematic monitoring of individuals with CH through registries and biorepositories, such as the UK Biobank and more recently the CHIVE biobank, is essential for patient stratification and the identification of optimal and personalised intervention [6]. However, the introduction of CH screening into routine clinical practice may present several limitations. These include the costs of the techniques, the need for specialised tools and personnel to interpret the data, and ethical implications which require clear consent processes. Moreover, there are no immediate clinical consequences, and it is not entirely clear which mutations or VAF thresholds should trigger clinical concern and action. It is expected that, in the future, routine CH screening may help stratify patients and guide the selection of personalised therapeutic strategies.

Interestingly, a combination of drugs such as metformin (a glucose‐lowering biguanide), nifedipine (a dihydropyrine calcium channel blocker), and MCC950 (an inflammasome inhibitor) have been shown to suppress CH‐mutant cell expansion by acting on different signalling pathways [33]. Despite being in use since 1957, the role of metformin in the context of CH‐associated diabetes is now increasingly recognised. The primary action of metformin is to lower blood glucose by acting on the mitochondrial complex I, resulting in the activation of AMP‐activated protein kinase (AMPK) [83]. As a downstream target of AMPK, TET2 undergoes phosphorylation that enhances its stability [29], providing a rationale for the use of metformin to restore TET2 function. A recent study reported that metformin reduced the competitive advantage of Dmnt3a HSPCs through an inhibition of complex I and a downregulation of genes involved in the mitochondrial oxidative phosphorylation and reversed aberrant epigenetic modification by increasing the methylation potential of Dmnt3a [84]. Furthermore, this drug exhibits immunomodulatory properties [85] and blunts NETosis in vitro and in vivo [86], suggesting that metformin may act on CH‐associated diabetes by modulating inflammation.

The mechanistic link between hyperglycemia‐induced myelopoiesis and impaired HSPC mobilisation (‘mobilopathy’) offers an additional explanation for the association between CH and adverse outcomes in T2D. Specifically, CH‐driven myeloid bias, combined with the shortage of circulating HSPCs observed in diabetes, may synergistically promote microvascular damage, cardiovascular complications, and increased mortality in these patients.

Overall, our understanding of the central role of remodelling of the haematopoietic system in diabetes and cardiometabolic diseases highlights CH as both a biomarker and a therapeutic target. This conceptual framework underscores the need to develop strategies to scale the detection of CH and to devise therapies aimed at modulating CH‐induced myelopoiesis and mitigate cardiometabolic complications.

## Author Contributions

Ludovica Migliozzi conceptualised, wrote, prepared the figures and reviewed the manuscript. Marella Marassi and Mattia Albiero corrected and reviewed the manuscript. Gian Paolo Fadini conceptualised, wrote, reviewed and gave the final approval.

## Funding

This work was supported by Ministero dell'Università e della Ricerca (European Union ‐ Next Generation EU, Mission 4, Component 1, CUP C53D23006470006, PRIN Grant 2022MZTHWJ).

## Conflicts of Interest

The authors declare no conflicts of interest.

## Data Availability

Data sharing is not applicable to this article as no new data were created or analysed in this study.
